# Co-consumption of Vegetables and Fruit, Whole Grains, and Fiber Reduces the Cancer Risk of Red and Processed Meat in a Large Prospective Cohort of Adults from Alberta’s Tomorrow Project

**DOI:** 10.3390/nu12082265

**Published:** 2020-07-29

**Authors:** Katerina Maximova, Elham Khodayari Moez, Julia Dabravolskaj, Alexa R. Ferdinands, Irina Dinu, Geraldine Lo Siou, Ala Al Rajabi, Paul J. Veugelers

**Affiliations:** 1 School of Public Health, University of Alberta, Edmonton, AB T6G 1C9, Canada; ekhodaya@ualberta.ca (E.K.M.); dabravol@ualberta.ca (J.D.); aferdina@ualberta.ca (A.R.F.); idinu@ualberta.ca (I.D.); Paul.Veugelers@ualberta.ca (P.J.V.); 2 Alberta’s Tomorrow Project, Cancer Research & Analytics, CancerControl Alberta, Alberta Health Services, Calgary, AB T2T 5C7, Canada; losioug@gmail.com; 3 Health Sciences Department, College of Natural and Health Sciences, Zayed University, Abu Dhabi 144534, UAE; ala.alrajabi@zu.ac.ae

**Keywords:** cancer prevention, red meat, processed meat, vegetables and fruit, whole grains, fiber, healthy eating

## Abstract

We examined whether co-consumption of red and processed meat with key foods items and food constituents recommended for cancer prevention (vegetables and fruit, whole grains, and fiber) mitigates cancer incidence. In a prospective cohort of 26,218 adults aged 35–69 years at baseline, dietary intake was collected through 124-item past-year food frequency questionnaire. Incidence of all-cause and 15 cancers previously linked to red and processed meat intake was obtained through data linkage with a cancer registry (average follow-up 13.5 years). Competing risk Cox Proportional Hazard models estimated cancer risk and Accelerated Failure Time models estimated time-to-cancer occurrence for different combinations of intake levels while considering mortality from vital statistics and established confounders. Co-consumption of low vegetables and fruit intake with high processed meat was associated with higher incidence of all-cause and 15 cancers (men: HR = 1.85, 1.91; women: HR = 1.44, 1.49) and accelerated time-to-cancer occurrence (men: 6.5 and 7.1 years and women: 5.6 and 6.3 years, respectively), compared to high vegetables and fruit with low processed meat intake. Less pronounced and less consistent associations were observed for whole grains and fiber and for red meat. The findings provide initial evidence toward refining existing cancer prevention recommendations to optimize the intake and combination of foods in the general adult population.

## 1. Introduction

Despite evidence that up to 35% of cancers are preventable by adherence to a healthy diet, maintaining a healthy weight and regular physical activity [1], the prevalence of these modifiable risk factors is high [2,3,4]. The World Cancer Research Fund/American Institute for Cancer Research (WCRF/AICR) recommend a healthy diet that is rich in vegetables, fruit, whole grains, and pulses; contains low amounts of red meat (i.e., meat that is red before being cooked (beef, veal, pork, mutton, lamb, horse, or goat) [5] and does not contain processed meats (ham, bacon, sausages, or meat that is transformed through curing, smoking, drying or other processes to improve the flavor or the quality, and may contain poultry, offal, or meat by-products) [6] as priorities for cancer prevention [1]. The inclusion of meat into the WCRF/AICR recommendations has sparked a debate regarding epidemiological evidence that implicates red and processed meat in carcinogenesis [7]. Potential mechanisms underlying the carcinogenesis of red and processed meat include *N*-nitroso compounds (NOC), heterocyclic amines (HCA), and polycyclic aromatic hydrocarbons (PAH), which are the mutagenic compounds formed during high-temperature cooking (frying, grilling, and barbecuing) and processing (curing and smoking) [8,9,10]. Following a review of scientific literature, the International Agency for Research on Cancer (IARC) later classified the consumption of red meat as probably carcinogenic and of processed meat as convincingly carcinogenic to humans [6,11]. The existing evidence focused on isolating the effect of individual food items and food constituents (i.e., red or processed meat) on cancer risk. Yet, it is being increasingly recognized that foods or nutrients are not consumed in isolation and may act synergistically to produce the effect from a combination of influences on several pathways involved in carcinogenesis [12]. In order to better capture the cancer risk associated with meat intake, there is a need to consider the role of potential synergies between the key food items and food constituents recommended for cancer prevention as part of a healthy diet. We examined the co-consumption of adverse intakes of red and processed meat, vegetables and fruit, whole grains, and fiber and its impact on cancer incidence and time-to-cancer occurrence in a prospective cohort study that follows a general provincial sample of Canadian adults.

## 2. Materials and Methods

Between 2001 and 2009, Alberta’s Tomorrow Project (ATP) recruited 31,072 adults through a two-stage probability sample of non-institutionalized individuals aged 35–69 years living in the province of Alberta, Canada, that were randomly selected in 8 waves of telephone-based random digit dialing (RDD) within regional health authority boundaries in Alberta as the sampling frame. Individuals that completed a Health and Lifestyle Questionnaire (HLQ) and signed a consent form were enrolled (49% of those contacted by RDD). Participants were excluded if they did not meet one of the following four criteria at enrollment: (i) aged 35–69 years; (ii) no prior personal history of cancer, other than non-melanoma skin cancer; (iii) plans to reside in Alberta for at least 1 year; and (iv) able to complete written questionnaires in English. Detailed protocols which describe all data collection procedures have been previously published [13,14].

For 30,431 (99%) participants that provided personal health numbers (PHN) at enrollment and consented to data linkage with administrative health databases, ATP data are linked on a regular basis (2 times/year) with the Alberta Cancer Registry (ACR) to determine the incidence of all types of cancer except non-melanoma skin cancer. The ACR is gold certified with the North American Association of Central Cancer Registries based on completeness and accuracy of the data [15]. All ATP records with valid PHN (100%) have been successfully linked. Cancer case ascertainment within ATP is high considering that very few participants (0.6%) have moved out of province since enrollment. There have been 2535 incident primary malignant all-cause cancers, excluding non-melanoma skin cancer, recorded up to December 2018 (average follow-up 13.5 years). Of these, 2208 cases were for 15 cancers with possible links to red and processed meat intake, including colorectal (CRC), stomach, pancreas, prostate, breast, bronchus/lung, esophagus, kidney, bladder, ovary, endometrium, non-Hodgkin lymphoma, liver and intrahepatic bile ducts, leukemia, and other (thyroid, gallbladder and biliary tract, testis, and brain) [11]. ATP data are also linked with the Vital Statistics to ascertain mortality from any cause. ATP study procedures and data linkage to the ACR and Vital Statistics (HREBA.CC-17–0461) and current analyses (HREBA.CC-17–0099) were approved by the Health Research Ethics Board of Alberta (HREBA)—Cancer Committee.

Dietary intake of red meat (g/day), processed meat (g/day), non-starchy vegetables and fruit excluding juices (servings/day), whole grains (servings/day), and fiber (g/day) was assessed by the Canadian Diet History Questionnaire I (CDHQ-I) that was administered 3 months after enrollment. The CDHQ-I is a 124-item past-year food frequency questionnaire (FFQ) of foods, beverages, and dietary supplements. The CDHQ-I is based on the Diet History Questionnaire (DHQ) developed by the US National Cancer Institute [16] and adapted for use in Canada to reflect food availability, brand names, nutrient composition, and food fortification [17,18]. The CDHQ-I food list has been shown to be representative of the foods commonly consumed by Canadian adults [17]. Responses to the CDHQ-I were analyzed using Diet*Calc (version 1.4.3, National Cancer Institute, Bethesda, MD, USA) with a nutrient database modified for the CDHQ-I, resulting in daily intake data for foods, food groups, energy, 66 nutrients, and supplements. The intakes were categorized into low, moderate, and high intake tertiles. The tertile cut-off values for vegetables and fruit and whole grains approximated established gender-specific dietary recommendations [19,20,21] and those for red and processed meat approximated the recommendations by the 2015 Global Burden Disease (GBD) [22] and WCRF/AICR [1].

Data analyses controlled for known confounders [12,23,24], including gender (men vs. women), smoking status (current smoker, former smoker, and never smoked based on the number of cigarettes smoked occasionally and daily for the past 30 days), alcohol use (number of drinks per day), first degree family (father, mother, and siblings) history of cancer (yes/no self-reported physician diagnosis), and personal history of chronic disease (yes/no self-reported physician diagnosis). Rural or urban residence at the time of enrollment was determined by postal reported weight divided by self-reported height squared and weight status categories (normal weight, overweight, and obese) were defined according to the WHO and Health Canada guidelines as BMI <25 code. Body Mass Index (BMI) was derived as self- ≥25 and ≥30 kg/m^2^, respectively. Underweight participants (0.7%) with BMI <18.5 kg/m^2^ were collapsed with the normal weight category to maximize sample size. Moderate and vigorous intensity recreational physical activity over the past year was assessed using a validated Past Year Total Physical Activity Questionnaire (PYTPAQ) [25]. Total minutes per week performing leisure activities at moderate (3–6 metabolic equivalent of tasks (METs)) and vigorous (>6 METs) intensities were calculated based on reported activities. Participants reported the highest level of education completed and the range of total gross household income. Analyses also adjusted for total energy intake (kcal per day) as recommended for analyses of FFQ data [26].

## 3. Data Analyses

Of 26,788 participants that completed the CDHQ-I, we excluded those who were recruited as “second in household” (*n* = 342), reported having a history of cancer prior to enrollment (*n* = 71), and did not give consent for linkage to the Alberta Cancer Registry (*n* = 180), resulting in 26,218 participants available for analysis (*n* = 23 met multiple exclusion criteria). To assess the effect of co-consumption of red and processed meat with varying levels of vegetables and fruit, whole grains, and fiber on the likelihood of developing cancer, we fitted competing risk Cox Proportional Hazard (PH) models to estimate the hazard ratios (HR) and 95% confidence intervals (95% CI) of cancer incidence for different combinations of low, moderate, and high intakes of dietary factors, using all-cause mortality as a competing risk [27,28,29]. The PH assumption was verified using a time interaction model and a modified Schoenfeld residual test. To account for the possibility that older participants had healthier diets at baseline and that analyses using Cox PH models may underestimate the effect of diet on cancer risk [30,31], we considered the survival times to be left-truncated at the age of enrollment. We also fitted Accelerated Failure Time (AFT) models to estimate the impact of a given combination of dietary factors on the median age of cancer occurrence [32]. The AFT is a powerful parametric method that can accommodate the non-PH assumption while taking into account differences in the age at enrollment. The AFT models’ goodness of fit was premised on the lognormal distribution of the error term and was evaluated using the Cox-Snell residuals plots, Q-Q plots, and AIC values [33,34,35].

All Cox PH models were adjusted for total energy intake, family history of cancer, personal history of chronic disease, rural/urban residence, smoking status, alcohol use, BMI, physical activity, and education. The AFT models were further adjusted for age at enrollment. The models were fitted separately for all-cause cancers and 15 cancers that have been linked to red and processed meat intake. Further, all analyses were stratified by gender since not all cancers affect both males and females (e.g., prostate cancer and ovarian cancer). To illustrate the synergistic role of different combinations of dietary factors, we adopted a novel post-estimation approach to present results as a matrix of nine *pairwise* linear combinations of coefficients for low, moderate, and high intakes, using the group with the healthiest diet as a reference (i.e., high intake of non-starchy vegetables and fruit, whole grains, or fiber with low intake of red meat or processed meat) [36].

Sensitivity analyses included models that additionally adjusted for total fat intake, dairy food or calcium intake, plant food or folate intake, and sodium, which have been shown to have protective effects in cancer etiology [12]. Sensitivity analyses also included models that (a) censored observations following diagnoses of other cancers (i.e., other than 15 cancers) [37], (b) excluded participants with extreme or unrealistic energy intakes (i.e., <500 and >5000 kcal per day) [26], (c) excluded participants diagnosed with any type of cancer in the first 2 years of follow-up as recommended for analyses of cancer cohort data [38,39], and (d) adjusted models with fiber intake for non-starchy vegetables and fruit. Finally, all models were repeated using gender-specific tertiles of dietary intakes without regard for established recommendations (Supplementary Tables S1 and S2). Since results remained robust in all sensitivity analyses, we present results from the most parsimonious models. Analyses were performed using SAS 9.4 and R 3.4.2, packages of cmprsk and survival.

## 4. Results

Participants were on average 50.4 (±9.2) years old, 59.9% were women, and 76.5% lived in urban areas (Table 1). Over half (52.5%) reported family history of cancer, almost half (46.8%) reported personal history of at least one chronic disease, 55.1% were either current or former smokers, and 65.7% were overweight and obese. Participants reported a wide range of household income and educational attainment, with nearly three-quarters (72.1%) having completed some postsecondary education. In terms of cancer incidence, 9.7% were diagnosed with cancer during follow-up, including 8.4% of 15 cancers, 1.5% of GI cancers, and 1.0% of CRC.

Men with low intake of vegetables and fruit combined with high intake of processed meat were 1.9 times as likely to develop cancer and 1.8 times as likely to develop one of 15 cancers during follow-up, compared to men with high intake of vegetables and fruit combined with low intake of processed meat (Table 2). The corresponding risk for women was 1.4 for all-cause cancers and 1.5 for 15 cancers (Table 3). Adherence to a diet high in vegetables and fruit and low in processed meat was associated with younger median age of all-cause cancers and 15 cancers for men by approximately 6.5 and 7.1 years (Table 4: 77.5 vs. 71.0 and 80.4 vs. 73.3), respectively. These differences in the estimated median age for women were 5.6 years for all-cause cancers and 6.3 years for 15 cancers (Table 5: 76.8 vs. 71.2 and 79.3 vs. 72.9).

Slightly weaker modification of cancer risk and time-to-cancer occurrence associated with processed meat intake was observed for fiber and whole grains. Weaker and less consistent modification of cancer incidence and time-to-cancer occurrence was observed for co-consumption of red meat with healthful dietary factors.

## 5. Discussion

In this prospective cohort study of a general provincial sample of Canadian adults, we examined the co-consumption of adverse intakes of red and processed meat, non-starchy vegetables and fruit, whole grains, and fiber, and its impact on cancer risk and time-to-cancer occurrence. Overall, low intake of vegetables and fruit was associated with higher incidence of all-cause and 15 cancers and accelerated time-to-cancer occurrence at any level of processed meat intake among both men and women. Results demonstrate that the carcinogenic effect of processed meat may be mitigated by following a healthy diet rich in non-starchy vegetables and fruit, particularly at low and moderate levels of processed meat intake. The consumption of these food items and food constituents varied between men and women, with men having substantially higher intakes of meat (both red and processed) and lower intakes of healthful food items and food constituents (vegetables and fruit, whole grains, and fiber). Men with adverse intakes of processed meat, vegetables and fruit, whole grains, and fiber were almost twice as likely to develop all-cause and 15 cancers compared to men with the healthiest intakes of these food items and food constituents, and cancer occurred 7 years earlier. Although the findings for red meat did not attain significance, they demonstrated a similar pattern.

Red meat serves as an important source of protein in Western diets and contains micronutrients (iron, selenium, zinc, omega-3 fatty acids, and vitamins B6, B12, A, D, and folic acid) and other bioactive components (e.g., taurine, creatine, carnosine, and glutathione) that are essential for human health [40]. Although earlier studies reported lower incidence of cancer for vegetarians compared to meat eaters [41], later studies demonstrated that low levels of meat consumption do not confer increased cancer risk [42,43], highlighting the importance of consuming meat as part of a varied and balanced diet. However, meat consumption in many North American and Western countries is very high [44] and well above the current WCRF/AICR recommendations [1]. For example, nationally representative data from the 2013–2014 and 2015–2016 National Health and Nutrition Examination Survey (NHANES) show that consumption of red meat among the US adults aged 20 years and older was, on average, 1.47 (SD = 0.43) servings per *day* compared to the optimal intake of 1.0 (SD = 0.1) serving per *week* recommended by WCRF/AICR, whereas consumption of processed meat was, on average, 0.87 (SD = 0.39) servings per day compared to no intake recommended by WCRF/AICR [45]. Similarly, Canada has one of the highest per capita meat consumption in the world at levels that are well above the WCRF/AICR recommendations [44] and 96% of Canadians consume meat as part of their diets [46]. Globally, the average per capita meat consumption has almost doubled from about 23 kg in 1961 to 43 kg in 2014, with consumption in high-income countries either static or declining [47,48]. Indeed, unprocessed red meat consumption in the US declined between 1999 and 2016, while consumption of processed red meat remained the same [49]. Similarly, the CCHS 24-h dietary recall data from 2004 to 2015 revealed that the intake of processed meat remained the same in all age groups [50]. The high intakes of meat, and processed meat in particular, may underlie the increasing trends in obesity-related cancers (notably, CRC, pancreas, endometrium, kidney, liver, prostate, thyroid, and ureter) in the past few decades, with increasing incidence observed in younger generations and age groups [51,52,53].

We observed strong associations for processed meat, however, the findings for red meat did not attain significance. This seems consistent with the existing evidence. Based on the strength of the epidemiological evidence, international expert reviews *convincingly* implicate processed meat consumption in cancer incidence, particularly diet- and hormone-related, while the role of red meat intake in carcinogenesis is graded as *probable* [1,6,11]. Indeed, published studies find stronger evidence for processed meat (CRC and stomach) vs. unprocessed red meat (CRC, pancreatic, and prostate) [43,54,55,56,57], and in North American vs. European studies [6,23,24], with the strongest evidence for CRC [58]. The evidence comes predominantly from case-control studies which are prone to recall bias (arising from retrospective collection of dietary exposures after the diagnosis) and selection bias (arising from inappropriate selection of controls), while the methodologically stronger prospective cohort studies in the general population are less consistent [6,59]. Although significant, the magnitude of risk associated with red and processed meat intake is generally weak (less than 20–30%). For example, meta-analyses estimate that the risk of CRC increases 12% for every 100 g of daily red meat intake and 17% for every 50 g of daily processed meat intake [24,60]. It has been suggested that the weak associations between red and processed meat consumption and cancer risk observed in prior studies may be confounded by other dietary factors [61,62,63], however, a few studies have considered the impact of co-consumption of dietary factors on cancer risk.

Nutritional epidemiology has traditionally focused on isolating the effect of a single food or nutrient on cancer risk. Yet, dietary factors are usually interrelated, and individuals consuming higher intakes of red and processed meat may also consume less fiber, vegetables, and fruit. Conversely, consumption of red or processed meat in combination with other foods rich in fiber, antioxidants, phytochemicals, calcium, and other nutrients, found in vegetables and fruit, whole grains, and pulses, may mitigate the carcinogenic effects of meat consumption [12]. Previous studies have adopted the dietary pattern approach, which considers the potential interaction between different dietary exposures. Several Canadian studies demonstrated that the Western dietary pattern (characterized by higher intakes of red and processed meat, highly processed and refined foods, salt, and sugar) is associated with higher cancer occurrence, recurrence, and mortality, compared to the “prudent” plant-based pattern [64,65,66]. However, the patterns are empirically derived using principal component factor analysis (PCFA) and therefore cannot delineate the effects of specific food items or food constituents recommended for cancer prevention as part of a healthy diet (e.g., red and processed meat, vegetables and fruit, and fiber) or be generalizable to other populations. In this study, we take a novel approach to consider the impact of co-consumption of adverse intakes of red and processed meat, vegetables and fruit, whole grains, and fiber on cancer risk. Our findings demonstrate that consumption of foods rich in fiber, antioxidants, phytochemicals, calcium, and other nutrients, found in vegetables and fruit, whole grains, and pulses, may have the potential to mitigate the carcinogenic effects of red and processed meat, particularly at lower and moderate—but not at higher—levels of meat intake. As the steepest increases in the risk of colorectal adenomas were reported at the lower levels of red and processed meat intake [23], these findings are pertinent to optimizing cancer prevention recommendations.

Although the substantial risk (OR = 2.0) for diet-related cancers (CRC, stomach, esophageal, and pancreatic) associated with low vegetables and fruit intake reported by early case-control studies was weaker in prospective cohort studies, the evidence continues to support a role of vegetables and fruit in carcinogenesis [60,67]. The protective effects of dietary fiber and whole grains are well established [68], explained by an increase in fecal bulk and decrease in intestinal transit time, thereby diluting carcinogens and reducing their absorption [69]. The associations between the intake of whole grains and cancer risk were less consistent in a systematic review of 20 longitudinal studies, with 6 studies reporting 6–47% reduction in cancer risk (CRC, upper digestive tract, renal, and head and neck cancers), while most studies (14 of 20) reported a null association between whole grains and cancer [69]. Our results for vegetables and fruit, whole grains, and fiber seem consistent with this evidence. While the associations of isolated dietary factors with cancer risk are valuable, previous studies did not examine the cumulative effect of several dietary factors recommended for cancer prevention simultaneously. As food items and food constituents are not consumed in isolation, research demonstrating the concurrent associations of co-consumption of adverse intakes of red and processed meat, vegetables and fruit, whole grains, and fiber with cancer risk is particularly pertinent. Our study makes a novel contribution to the literature by demonstrating a substantially higher risk associated with co-consumption of multiple adverse intakes of dietary food items and food constituents recommended for cancer prevention, likely suggesting that the carcinogenic effect may be cumulative.

We observed differences in the observed associations between men and women. According to the 2004 Canadian Community Health Survey (CCHS) based on the 24-h dietary recall from a nationally representative sample of 35,107 Canadians, men and women aged 19 years and older consumed, on average, 702 and 386 g per week of red meat (both unprocessed and processed), of which 193 and 106 g per week, respectively, is processed [70]. In addition, considerably more Canadian men than women (25% vs. 14%) consume 500 or more grams of red and processed meat weekly [71]. Although a few studies reported sex patterning in the associations of red and processed meat consumption with cancer risk [67], the incidence and population attributable fractions of diet-related cancers are higher among men than women [45]. Our findings demonstrate that men are at a greater risk of all-cause and 15 cancers compared to women, and cancer occurs earlier in men than in women.

The large sample of the general adult population of Alberta with a diverse range of demographic and behavioral characteristics, the nearly complete linkage with administrative databases, and little missing data due to rigorous quality control measures are major strengths. Several limitations warrant consideration. Dietary intake data were collected through self-report FFQ, which are prone to measurement error and may not accurately capture the absolute level of food intake. However, FFQ is the most commonly used and feasible method of dietary assessment in cohort studies of cancer risk [72,73]. We adjusted for total energy intake to take into account the absolute level of food intake [26]. Dietary intake reported at enrollment may not be a reliable estimation of habitual diet over the course of follow-up. However, prior cohort studies suggest that dietary habits remain relatively stable during adulthood [74,75]. Data on important confounders (smoking and alcohol use) were collected through self-report and residual confounding may persist despite adjustment. However, the validity of self-report smoking is consistently high in population-based studies [76]. Another limitation is related to the ascertainment of incident cases in administrative data that can be affected by delays along the cancer diagnostic pathway, including patient (e.g., poor recognition of early symptoms), doctor (e.g., the lack of recognition, investigation, and referral for suspicious symptoms), and system (e.g., delays in initiating treatment) factors [77]. Lastly, the small number of incident cases precluded assessment of the associations separately for CRC and gastrointestinal cancers.

Researchers have only recently begun to examine the presence of interactions between different food items and their relation to cancer risk [78]. The recommendation to limit the intake of red meat and avoid processed meat has high relevance for public health, given high levels of meat consumption in Western countries [44] and the high cancer burden attributable to red and processed meat consumption in Canada [79]. The results from this study provide new knowledge about the potential synergies of dietary intakes of red and processed meat, vegetables and fruit, whole grains, and fiber for the development of cancer risk and time-to-cancer occurrence. Results underscore the benefits of non-starchy vegetables and fruit on mitigating cancer risk and decelerating time-to-cancer occurrence. The findings inform existing recommendations to optimize the intake and combination of foods in the general adult population and provide direction for future prevention efforts to focus on promoting the intake of non-starchy vegetables and fruit when consuming meat.

## Figures and Tables

**Table 1:** Baseline characteristics of Alberta’s Tomorrow Project participants by gender (*n* = 26,218).

	Men	Women	Total
	(*n* = 9825)	(*n* = 16,393)	(*n* = 26,218)
	% or Mean (SD)	% or Mean (SD)	% or Mean (SD)
Age at enrollment, years	50.5 (9.2)	50.3 (9.2)	50.4 (9.2)
Family history of cancer *	50.2	53.9	52.5
Personal history of chronic disease * ^a^	50.6	44.6	46.8
Geographic location *			
Urban	77.5	75.9	76.5
Rural	22.5	24.1	23.5
Educational attainment *			
High school or less	25.0	29.8	28.0
Technical, college, some university	47.2	46.7	46.8
University and postgraduate	27.8	23.7	25.3
Annual household income *			
≤$39,999	15.4	24.5	21.7
$40,000–$69,999	28.1	27.2	27.6
≥$70,000	55.1	44.4	48.4
Smoking status *			
Current smoker	18.1	17.2	17.5
Former smoker	39.8	36.3	37.6
Never smoked	42.0	46.5	44.8
Alcohol intake, drinks/day *	1.3 (3.4)	0.5 (1.5)	0.8 (2.4)
Weight status *			
Normal weight (<25 kg/m^2^)	23.1	39.3	34.0
Overweight (25–30 kg/m^2^)	49.7	33.7	39.7
Obese (>30 kg/m^2^)	27.0	25.5	26.0
Body Mass Index, kg/m^2^*	28.1 (4.4)	27.3 (5.9)	27.6 (5.4)
Moderate or vigorous physical activity, MET-hours/week * ^b^	20.4 (24.8)	15.5 (20.9)	17.3 (22.6)
Total energy intake, kcal/day *	2235 (1017)	1641 (668)	1863 (866)
Dietary intake			
Red meat, gram/week *	461.4 (347.5)	262.9 (191.9)	337.3 (278.4)
Processed meat, gram/week *	172.6 (170.4)	85.9 (93.3)	118.4 (134.5)
Vegetables and fruit, serving/day *	4.3 (2.9)	4.9 (3.2)	4.7 (3.1)
Whole grains, serving/day *	1.3 (1.0)	1.0 (0.7)	1.1 (0.8)
Fiber, gram/week *	157.3 (71.5)	136 (61.4)	144 (61.1)
Cancer incidence			
All-cause cancers * ^c^	11.0	8.9	9.7
15 cancers* ^d^	9.6	7.7	8.4
Gastrointestinal (GI) cancers * ^e^	2.0	1.3	1.5
Colorectal cancers * ^f^	1.2	0.9	1.0
All-cause mortality *	3.7	2.4	2.9

SD, standard deviation. Notes: The percentages do not sum up to 100 due to missing values. * *p* < 0.05 for men vs. women based on a Pearson chi-squared test for categorical variables and a two-sample *t*-test for continuous variables. ^a^ Includes high blood pressure, angina, high cholesterol in blood, heat attack, stroke, emphysema, chronic bronchitis, diabetes, polyps in colon or rectum, ulcerative colitis, Crohn’s disease, hepatitis, and cirrhosis of liver. ^b^ Total metabolic equivalent (MET)-hours per week spent performing recreational physical activities at moderate (>3 to ≤6 MET) or vigorous (>6 MET) intensity. ^c^ Primary malignant cancers, excluding nonmelanoma skin cancer. ^d^ Overall, 15 cancers with possible links to red and processed meat intake, including colorectal, stomach, pancreas, prostate, breast, bronchus/lung, esophagus, kidney, bladder, ovary, endometrium, non-Hodgkin lymphoma, liver and intrahepatic bile ducts, leukemia, and other (thyroid, gallbladder and biliary tract, testis, and brain) [11]. ^e^ Esophagus, stomach, small intestine, colon, rectum and rectosigmoid, anus, anal canal and anorectum, liver and intrahepatic bile ducts, gallbladder and extrahepatic bile ducts, biliary tract, and exocrine pancreas. ^f^ Colon, rectum, rectosigmoid, and non-Hodgkin lymphoma.

**Table 2:** Estimated hazard ratios ^a^ and confidence intervals for all-cause and 15 cancer incidence for different combinations of intake levels of red and processed meat with vegetables and fruit, whole grains, and fiber among men, Alberta’s Tomorrow Project (*n* = 9825).

	All-Cause Cancers ^b^			15 Cancers ^c^		
	Vegetables and Fruit (Serving/Day) ^d^			Vegetables and Fruit (Serving/Day) ^d^		
	<55 years: <4	<55 years: 4–6	<55 years: >6	<55 years: <4	<55 years: 4–6	<55 years: >6
	≥55 years: <3	≥55 years: 3–5	≥55 years: >5	≥55 years: <3	≥55 years: 3–5	≥55 years: >5
Red meat (gram/week) ^e^						
<250	1.04 (0.79–1.36)	1.02 (0.89–1.17)	Ref.	0.97 (0.73–1.30)	0.99 (0.85–1.14)	Ref.
250–500	1.17 (0.92–1.47)	1.01 (0.85–1.21)	0.88 (0.76–1.02)	1.18 (0.92–1.52)	1.01 (0.84–1.22)	0.86 (0.73–1.02)
>500	**1.31 (1.02–1.69)**	1.01 (0.79–1.29)	0.78 (0.57–1.05)	**1.44 (1.10–1.88)**	1.04 (0.79–1.35)	0.75 (0.54–1.04)
Processed meat (gram/week) ^f^						
<42	**1.56 (1.13–2.16)**	**1.25 (1.06–1.47)**	Ref.	**1.53 (1.08–2.16)**	**1.24 (1.04–1.47)**	Ref.
42–168	**1.73 (1.32–2.26)**	**1.49 (1.22–1.83)**	**1.29 (1.09–1.52)**	**1.68 (1.26–2.24)**	**1.43 (1.15–1.77)**	**1.21 (1.02–1.45)**
>168	**1.91 (1.45–2.51)**	**1.78 (1.35–2.35)**	**1.66 (1.19–2.31)**	**1.85 (1.38–2.48)**	**1.65 (1.23–2.22)**	**1.47 (1.04–2.09)**
	Whole grains (serving/day) ^g^			Whole grains (serving/day) ^g^		
	<0.75	0.75–1.5	>1.5	<0.75	0.75–1.5	>1.5
Red meat (gram/week)						
<250	1.22 (0.94–1.58)	1.11 (0.972–1.26)	Ref.	1.30 (0.99–1.71)	1.14 (0.99–1.31)	Ref.
250–500	1.10 (0.90–1.36)	1.12 (0.96–1.31)	1.14 (1.00–1.30)	1.25 (1.00–1.56)	**1.20 (1.02–1.42)**	**1.17 (1.01–1.34)**
>500	1.00 (0.79–1.27)	1.14 (0.92–1.41)	1.30 (1.00–1.68)	1.19 (0.92–1.54)	**1.27 (1.01–1.61)**	**1.36 (1.03–1.80)**
Processed meat (gram/week)						
<42	0.90 (0.67–1.22)	0.95 (0.82–1.10)	Ref.	0.91 (0.66–1.26)	0.95 (0.81–1.12)	Ref.
42–168	1.11 (0.87–1.40)	1.13 (0.95–1.35)	**1.16 (1.01–1.33)**	1.14 (0.88–1.47)	1.11 (0.92–1.34)	1.09 (0.93–1.26)
>168	**1.36 (1.05–1.75)**	**1.35 (1.06–1.72)**	**1.34 (1.02–1.77)**	**1.41 (1.08–1.86)**	1.29 (0.99–1.68)	1.18 (0.87–1.60)
	Fiber (gram/week) ^h^			Fiber (gram/week) ^h^		
	<117	117–150	>150	<117	117–150	>150
Red meat (gram/week)						
<250	**1.39 (1.10–1.77)**	**1.18 (1.05–1.33)**	Ref.	1.24 (0.96–1.60)	1.11 (0.98–1.27)	Ref.
250–500	**1.28 (1.04–1.58)**	**1.18 (1.02–1.37)**	1.09 (0.97–1.23)	**1.33 (1.07–1.66)**	**1.19 (1.02–1.38)**	1.06 (0.93–1.20)
>500	1.18 (0.90–1.539)	1.19 (0.96–1.46)	1.19 (0.94–1.52)	**1.43 (1.08–1.90)**	**1.26 (1.01–1.58)**	1.11 (0.86–1.44)
Processed meat (gram/week)						
<42	**1.42 (1.07–1.89)**	**1.19 (1.03–1.37)**	Ref.	1.30 (0.96–1.76)	1.14 (0.98–1.33)	Ref.
42–168	**1.53 (1.22–1.92)**	**1.40 (1.19–1.65)**	**1.27 (1.12–1.45)**	**1.47 (1.15–1.87)**	**1.31 (1.10–1.56)**	**1.17 (1.02–1.35)**
>168	**1.65 (1.28–2.14)**	**1.64 (1.30–2.06)**	**1.62 (1.25–2.11)**	**1.66 (1.26–2.18)**	**1.51 (1.18–1.93)**	**1.38 (1.04–1.82)**

^a^ Adjusted for total energy intake, family history of cancer, personal history of chronic disease, rural/urban residence, smoking status, alcohol use, Body Mass Index, physical activity, and education. ^b^ Primary malignant cancers, excluding non-melanoma skin cancer. ^c^ Overall, 15 cancers with possible links to red and processed meat intake, including colorectal, stomach, pancreas, prostate, breast, bronchus/lung, esophagus, kidney, bladder, ovary, endometrium, non-Hodgkin lymphoma, liver and intrahepatic bile ducts, leukemia, and other (thyroid, gallbladder and biliary tract, testis, and brain) [11]. ^d^ The cut-off values for the middle and highest tertiles were chosen such that the median intake in the highest tertile approximated the Canada’s Food Guide recommendations of 8 servings per day for men >55 years old and 7 servings for men 55 years old and over [19], and the median intake in the middle tertile approximated the Canadian Cancer Society recommendations of 5 servings [20]. ^e^ The cut-off values for the tertiles were guided by the WCRF/AICR recommendations of 350–500 g per week [1], Canadian Cancer Society recommendations of 250 g per week (3 portions of <85 g) [20], and Global Burden of Disease recommendations of 126–189 g per week (8–27 g per day) [22]. ^f^ The cut-off value for the lowest tertile was guided by the recommendation of half a serving of 85 g per week as per Dietary Guidelines for Americans [21]. ^g^ As the consumption levels for whole grains were substantially lower than the existing recommendations (e.g., Canada’s Food Guide recommends 4 servings per day) [19], the cut-off values for the tertiles were guided by the actual consumption. ^h^ The cut-off values for the middle and highest tertiles were chosen such that the median intake approximated the Global Burden of Disease and Dietary Guidelines for Americans recommendations of 133 and 196 g per week (19 and 28 g per day) [21,22]. Bolded numbers indicate statistical significance at *p* < 0.05.

**Table 3:** Estimated hazard ratios ^a^ and confidence intervals for all-cause and 15 cancer incidence for different combinations of intake levels of red and processed meat with vegetables and fruit, whole grains, and fiber among women, Alberta’s Tomorrow Project (*n* = 16,393).

	All-Cause Cancers ^b^			15 Cancers ^c^		
	Vegetables and Fruit (Serving/Day) ^d^			Vegetables and Fruit (Serving/Day) ^d^		
	<3	3–5	>5	<3	3–5	>5
Red meat (gram/week) ^e^						
<150	1.02 (0.83–1.25)	1.01 (0.91–1.12)	Ref.	0.94 (0.76–1.17)	0.97 (0.87–1.08)	Ref.
150–300	1.06 (0.90–1.27)	1.00 (0.88–1.13)	0.93 (0.84–1.04)	1.02 (0.84–1.22)	0.96 (0.83–1.09)	0.90 (0.80–1.01)
>300	1.12 (0.91–1.37)	0.99 (0.82–1.18)	0.87 (0.70–1.08)	1.09 (0.87–1.37)	0.94 (0.78–1.14)	0.81 (0.64–1.02)
Processed meat (gram/week) ^f^						
<28	1.13 (0.90–1.43)	1.06 (0.95–1.20)	Ref.	1.07 (0.84–1.38)	1.036 (0.91–1.17)	Ref.
28–112	**1.28 (1.07–1.53)**	**1.21 (1.06–1.39)**	**1.15 (1.02–1.29)**	**1.26 (1.04–1.54)**	**1.21 (1.05–1.40)**	**1.16 (1.02–1.31)**
>112	**1.44 (1.17–1.77)**	**1.38 (1.14–1.67)**	**1.32 (1.05–1.67)**	**1.49 (1.19–1.86)**	**1.41 (1.15–1.73)**	**1.34 (1.04–1.72)**
	Whole grains (serving/day) ^g^			Whole grains (serving/day) ^g^		
	<0.6	0.6–1.1	>1.1	<0.6	0.6–1.1	>1.1
Red meat (gram/week)						
<150	0.90 (0.73–1.10)	0.95 (0.86–1.05)	Ref.	1.00 (0.81–1.25)	1.00 (0.90–1.12)	Ref.
150–300	0.95 (0.80–1.12)	0.93 (0.82–1.05)	0.92 (0.82–1.02)	1.06 (0.88–1.26)	0.97 (0.85–1.11)	0.90 (0.80–1.01)
>300	1.00 (0.82–1.21)	0.91 (0.77–1.09)	0.84 (0.68–1.05)	1.11 (0.91–1.37)	0.95 (0.78–1.14)	0.81 (0.64–1.02)
Processed meat (gram/week)						
<28	0.94 (0.75–1.18)	0.97 (0.87–1.08)	Ref.	1.07 (0.83–1.36)	1.03 (0.91–1.17)	Ref.
28–112	1.12 (0.94–1.35)	1.11 (0.97–1.27)	1.10 (0.97–1.23)	**1.30 (1.07–1.58)**	**1.21 (1.05–1.40)**	1.12 (0.99–1.27)
>112	**1.34 (1.09–1.66)**	**1.27 (1.05–1.54)**	1.20 (0.95–1.52)	**1.59 (1.27–1.99)**	**1.42 (1.16–1.74)**	1.26 (0.98–1.62)
	Fiber (gram/week) ^h^			Fiber (gram/week) ^h^		
	<110	110–161	>161	<110	110–161	>161
Red meat (gram/week)						
<150	1.11 (0.88–1.39)	1.05 (0.94–1.18)	Ref.	1.01 (0.79–1.30)	1.01 (0.89–1.14)	Ref.
150–300	1.13 (0.93–1.38)	1.02 (0.89–1.18)	0.93 (0.83–1.04)	1.03 (0.84–1.28)	0.98 (0.84–1.13)	0.93 (0.82–1.05)
>300	1.15 (0.93–1.43)	1.00 (0.83–1.21)	0.87 (0.69–1.09)	1.05 (0.83–1.33)	0.95 (0.78–1.17)	0.861 (0.67–1.10)
Processed meat (gram/week)						
<28	1.27 (0.99–1.62)	1.13 (1.00–1.27)	Ref.	1.1 (0.85–1.43)	1.05 (0.92–1.20)	Ref.
28–112	**1.38 (1.12–1.69)**	**1.28 (1.11–1.49)**	**1.19 (1.05–1.36)**	**1.26 (1.01–1.57)**	**1.228 (1.05–1.44)**	**1.20 (1.04–1.38)**
>112	**1.50 (1.20–1.87)**	**1.46 (1.19–1.79)**	**1.43 (1.10–1.85)**	**1.44 (1.14–1.82)**	**1.44 (1.15–1.79)**	**1.433 (1.08–1.90)**

^a^ Adjusted for total energy intake, smoking status, alcohol use, family history of cancer, personal history of chronic disease, rural/urban residence, Body Mass Index, physical activity, and education. ^b^ Primary malignant cancers, excluding non-melanoma skin cancer. ^c^ Overall, 15 cancers with possible links to red and processed meat intake, including colorectal, stomach, pancreas, prostate, breast, bronchus/lung, esophagus, kidney, bladder, ovary, endometrium, non-Hodgkin lymphoma, liver and intrahepatic bile ducts, leukemia, and other (thyroid, gallbladder and biliary tract, testis, and brain) [11]. ^d^ The cut-off value for the highest tertile was chosen such that the median intake in the highest tertile approximated the Canada’s Food Guide recommendations of 7 servings per day [19]. ^e^ The cut-off values for the tertiles were chosen such that the median intake approximated the WCRF/AICR recommendations of 350–500 g per week [1], Canadian Cancer Society recommendations of 250 g per week (3 portions of <85 g) [20], and Global Burden of Disease recommendations of 126–189 g per week (8–27 g per day) [22]. ^f^ The cut-off value for the lowest tertile was guided by the Global Burden of Disease recommendations of 28 g per week (0–4 g per day) [22]. ^g^ As the consumption levels for whole grains were substantially lower than the existing recommendations (e.g., Canada’s Food Guide recommends 4 servings per day) [19], the cut-off values for the tertiles were guided by the actual consumption. ^h^ The cut-off values for the middle and highest tertiles were chosen such that the median intake approximated the Global Burden of Disease and Dietary Guidelines for Americans recommendations of 133 and 196 g per week (19 and 28 g per day) [21,22]. Bolded numbers indicate statistical significance at *p* < 0.05.

**Table 4:** Estimated median ages ^a^ and confidence intervals for all-cause and 15 cancer incidence for different combinations of intake levels of red and processed meat with vegetables and fruit, whole grains, and fiber among men, Alberta’s Tomorrow Project (*n* = 9825).

	All-Cause Cancers ^b^			15 Cancers ^c^		
	Vegetables and Fruit (Serving/Day) ^d^			Vegetables and Fruit (Serving/Day) ^d^		
	<55 years: <4	<55 years: 4–6	<55 years: >6	<55 years: <4	<55 years: 4–6	<55 years: >6
	≥55 years: <3	≥55 years: 3–5	≥55 years: >5	≥55 years: <3	≥55 years: 3–5	≥55 years: >5	
Red meat (gram/week) ^e^						
<250	72.55 (70.80–74.34)	75.82 (73.68–78.02)	75.82 (73.51–78.21)	75.10 (73.08–77.18)	78.52 (76.03–81.08)	77.93 (75.29–80.66)
250–500	71.20 (69.61–72.83)	74.05 (72.22–75.93)	74.4 (72.37–76.49)	73.64 (71.79–75.54)	76.19 (74.09–78.36)	77.62 (75.20–80.11)
>500	**70.34 (68.66–72.05)**	74.16 (72.17–76.20)	75.08 (73.05–77.16)	**72.21 (70.30–74.17)**	77.09 (74.75–79.51)	77.84 (75.48–80.27)
Processed meat (gram/week) ^f^						
<42	**73.59 (71.36–75.89)**	**76.28 (73.53–79.12)**	77.57 (74.51–80.75)	**75.99 (73.41–78.66)**	**78.06 (74.99–81.25)**	80.37 (76.80–84.10)
42–168	**71.00 (69.49–72.53)**	**74.59 (72.82–76.40)**	**75.31 (73.37–77.30)**	**73.34 (71.60–75.13)**	**76.87 (74.83–78.97)**	**78.32 (76.04–80.67)**
>168	**71.03 (69.38–72.73)**	**73.81 (71.93–75.73)**	**73.81 (71.90–75.77)**	**73.26 (71.35–75.22)**	**77.02 (74.79–79.32)**	**76.44 (74.22–78.73)**
	Whole grains (serving/day) ^g^			Whole grains (serving/day) ^g^		
	<0.75	0.75–1.5	>1.5	<0.75	0.75–1.5	>1.5
Red meat (gram/week)						
<250	73.48 (71.52–75.50)	75.12 (73.01–77.29)	75.38 (73.35–77.46)	76.19 (73.91–78.54)	77.90 (75.44–80.43)	77.74 (75.41–80.14)
250–500	72.23 (70.34–74.17)	73.17 (71.40–74.97)	73.62 (71.86–75.43)	74.66 (72.48–76.90)	**75.60 (73.56–77.70)**	**76.56 (74.47–78.70)**
>500	72.55 (70.62–74.53)	71.93 (70.00–73.91)	73.96 (72.11–75.86)	74.59 (72.40–76.85)	**74.67 (72.42–77.00)**	**76.56 (74.40–78.78)**
Processed meat (gram/week)						
<42	75.01 (72.46–77.64)	76.78 (73.94–79.74)	75.65 (73.10–78.29)	77.79 (74.82–80.87)	79.06 (75.80–82.46)	77.67 (74.78–80.66)
42–168	72.80 (71.06–74.58)	73.09 (71.41–74.80)	**73.78 (72.08–75.52)**	75.08 (73.07–77.13)	75.70 (73.75–77.71)	76.43 (74.44–78.47)
>168	**71.30 (69.44–73.22)**	**72.32 (70.44–74.24)**	**74.00 (72.26–75.79)**	**73.61 (71.47–75.82)**	74.98 (72.79–77.23)	76.87 (74.82–78.98)
	Fiber (gram/week) ^h^			Fiber (gram/week) ^h^		
	<117	117–150	>150	<117	117–150	>150
Red meat (gram/week)						
<250	**73.85 (71.90–75.84)**	**73.40 (71.11–75.75)**	75.93 (73.89–78.02)	76.26 (74.02–78.57)	76.04 (73.4–78.78)	78.15 (75.82–80.55)
250–500	**72.52 (70.56–74.54)**	**72.65 (70.71–74.65)**	73.44 (71.8–75.11)	**74.93 (72.66–77.27)**	**74.72 (72.5–77)**	76.19 (74.27–78.15)
>500	71.72 (69.35–74.18)	71.9 (69.69–74.17)	73.69 (72.00–75.41)	**73.25 (70.62–75.99)**	**73.5 (71.04–76.05)**	76.72 (74.74–78.76)
Processed meat (gram/week)						
<42	**75.53 (72.93–78.21)**	**72.66 (69.87–75.57)**	77.68 (75.05–80.40)	78.43 (75.38–81.60)	74.10 (70.99–77.34)	79.80 (76.82–82.9)
42–168	**72.35 (70.58–74.16)**	**73.08 (71.21–75.01)**	**73.86 (72.26–75.49)**	**74.34 (72.32** **–76.41)**	**75.56 (73.39** **–77.79)**	**76.63 (74.75** **–78.55)**
>168	**72.55 (70.32–74.85)**	**71.77 (69.68–73.93)**	**73.20 (71.59–74.86)**	**75.1 (72.52** **–77.78)**	**73.56 (71.20** **–76.00)**	**76.17 (74.26** **–78.12)**

^a^ Adjusted for total energy intake, family history of cancer, personal history of chronic disease, rural/urban residence, smoking status, alcohol use, Body Mass Index, physical activity, education, and age at enrollment. ^b^ Primary malignant cancers, excluding non-melanoma skin cancer. ^c^ Overall, 15 cancers with possible links to red and processed meat intake, including colorectal, stomach, pancreas, prostate, breast, bronchus/lung, esophagus, kidney, bladder, ovary, endometrium, non-Hodgkin lymphoma, liver and intrahepatic bile ducts, leukemia, and other (thyroid, gallbladder and biliary tract, testis, and brain) [11]. ^d^ The cut-off values for the middle and highest tertiles were chosen such that the median intake in the highest tertile approximated the Canada’s Food Guide recommendations of 8 servings per day for men >55 years old and 7 servings for men 55 years old and over [19], and the median intake in the middle tertile approximated the Canadian Cancer Society recommendations of 5 servings [20]. ^e^ The cut-off values for the tertiles were guided by the WCRF/AICR recommendations of 350–500 g per week [1], Canadian Cancer Society recommendations of 250 g per week (3 portions of <85 g) [20], and Global Burden of Disease recommendations of 126–189 g per week (8–27 g per day) [22]. ^f^ The cut-off value for the lowest tertile was guided by the recommendation of half a serving of 85 g per week as per Dietary Guidelines for Americans [21]. ^g^ As the consumption levels for whole grains were substantially lower than the existing recommendations (e.g., Canada’s Food Guide recommends 4 servings per day) [19], the cut-off values for the tertiles were guided by the actual consumption. ^h^ The cut-off values for the middle and highest tertiles were chosen such that the median intake approximated the Global Burden of Disease and Dietary Guidelines for Americans recommendations of 133 and 196 g per week (19 and 28 g per day) [21,22]. Bolded numbers indicate statistical significance at *p* < 0.05.

**Table 5:** Estimated median ages ^a^ and 95% confidence intervals for all-cause and 15 cancer incidence for different combinations of intake levels of red and processed meat with vegetables and fruit, whole grains, and fiber among women, Alberta’s Tomorrow Project (*n* = 9825).

	All-Cause Cancers ^b^			15 Cancers ^c^		
	Vegetables and Fruit (Serving/Day) ^d^			Vegetables and Fruit (Serving/Day) ^d^		
	<3	3–5	>5	<3	3–5	>5
Red meat (gram/week) ^e^						
<150	74.04 (72.15–75.98)	77.28 (75.28–79.34)	75.75 (73.97–77.56)	76.93 (74.73–79.20)	79.50 (77.22–81.84)	77.74 (75.72–79.81)
150–300	73.21 (71.34–75.14)	75.55 (73.83–77.31)	77.02 (75.31–78.77)	75.45 (73.30–77.66)	77.84 (75.88–79.86)	79.97 (77.96–82.03)
>300	72.42 (70.30–74.59)	74.93 (73.19–76.72)	77.57 (75.75–79.44)	74.43 (72.04–76.89)	77.72 (75.67–79.83)	80.02 (77.92–82.17)
Processed meat (gram/week) ^f^						
<28	76.23 (73.94–78.59)	77.26 (75.18–79.39)	76.80 (74.96–78.69)	79.77 (77.04–82.59)	79.60 (77.23–82.05)	79.27 (77.14–81.46)
28–112	**73.31 (71.61–75.05)**	**76.35 (74.74–78.00)**	**76.89 (75.27–78.55)**	**75.73 (73.77–77.74)**	**78.98 (77.10–80.9)**	**79.23 (77.36–81.14)**
>112	**71.24 (69.17–73.37)**	**73.54 (71.66–75.48)**	**76.51 (74.64–78.43)**	**72.95 (70.63–75.34)**	**75.74 (73.58–77.95)**	**79.15 (76.97–81.38)**
	Whole grains (serving/day) ^g^			Whole grains (serving/day) ^g^		
	<0.6	0.6–1.1	>1.1	<0.6	0.6–1.1	>1.1
Red meat (gram/week)						
<150	75.38 (73.50–77.31)	76.61 (74.67–78.60)	75.77 (73.93–77.66)	77.81 (75.64–80.04)	78.89 (76.67–81.18)	78.06 (75.95–80.23)
150–300	74.53 (72.73–76.37)	75.66 (73.89–77.47)	76.82 (75.08–78.60)	76.93 (74.85–79.06)	77.92 (75.89–80)	79.61 (77.58–81.69)
>300	73.57 (71.70–75.49)	75.54 (73.66–77.47)	77.30 (75.45–79.19)	75.58 (73.45–77.76)	78.24 (76.04–80.49)	80.00 (77.86–82.20)
Processed meat (gram/week)						
<28	76.44 (74.43–78.51)	78.12 (75.97–80.33)	76.87 (74.87–78.92)	79.28 (76.92–81.70)	80.47 (78.01–83.00)	79.65 (77.33–82.05)
28–112	74.56 (72.90–76.26)	75.87 (74.23–77.56)	77.24 (75.60–78.92)	**76.91 (75–78.87)**	**78.26 (76.36–80.20)**	79.88 (77.97–81.83)
>112	**72.77 (70.75–74.85)**	**74.19 (72.23–76.21)**	75.41 (73.57–77.30)	**74.49 (72.23–76.83)**	**76.72 (74.45–79.06)**	77.78 (75.66–79.96)
	Fiber (gram/week) ^h^			Fiber (gram/week) ^h^		
	<110	110–161	>161	<110	110–161	>161
Red meat (gram/week)						
<150	74.13 (72.31–76.00)	74.73 (72.88–76.64)	76.75 (74.66–78.88)	77.06 (74.94–79.25)	76.78 (74.67–78.96)	78.87 (76.50–81.31)
150–300	72.56 (70.84–74.32)	75.58 (73.88–77.31)	77.99 (76.04–79.99)	75.05 (73.06–77.09)	78.17 (76.21–80.19)	80.67 (78.41–83)
>300	72.75 (70.72–74.84)	74.44 (72.73–76.19)	78.77 (76.78–80.81)	75.24 (72.9–77.66)	77.06 (75.08–79.11)	81.11 (78.83–83.46)
Processed meat (gram/week)						
<28	75.51 (73.49–77.60)	75.71 (73.72–77.75)	77.76 (75.61–79.97)	78.85 (76.45–81.33)	78.18 (75.89–80.54)	80.14 (77.68–82.68)
28–112	**73.62 (71.97–75.30)**	**75.22 (73.66–76.82)**	**78.03 (76.21–79.90)**	**76.44 (74.51–78.42)**	**77.50 (75.70–79.35)**	**80.55 (78.44–82.72)**
>112	**70.21 (68.26–72.22)**	**74.16 (72.29–76.06)**	**77.60 (75.54–79.72)**	**71.96 (69.77–74.22)**	**77.03 (74.85–79.27)**	**79.71 (77.36–82.14)**

^a^ Adjusted for total energy intake, smoking status, alcohol use, family history of cancer, personal history of chronic disease, rural/urban residence, Body Mass Index, physical activity, education, and age at enrollment. ^b^ Primary malignant cancers, excluding non-melanoma skin cancer. ^c^ Overall, 15 cancers with possible links to red and processed meat intake, including colorectal, stomach, pancreas, prostate, breast, bronchus/lung, esophagus, kidney, bladder, ovary, endometrium, non-Hodgkin lymphoma, liver and intrahepatic bile ducts, leukemia, and other (thyroid, gallbladder and biliary tract, testis, and brain) [11]. ^d^ The cut-off value for the highest tertile was chosen such that the median intake in the highest tertile approximated the Canada’s Food Guide recommendations of 7 servings per day [19]. ^e^ The cut-off values for the tertiles were chosen such that the median intake approximated the WCRF/AICR recommendations of 350–500 g per week [1], Canadian Cancer Society recommendations of 250 g per week (3 portions of <85 g) [20], and Global Burden of Disease recommendations of 126–189 g per week (8–27 g per day) [22]. ^f^ The cut-off value for the lowest tertile was guided by the Global Burden of Disease recommendations of 28 g per week (0–4 g per day) [22]. ^g^ As the consumption levels for whole grains were substantially lower than the existing recommendations (e.g., Canada’s Food Guide recommends 4 servings per day) [19], the cut-off values for the tertiles were guided by the actual consumption. ^h^ The cut-off values for the middle and highest tertiles were chosen such that the median intake approximated the Global Burden of Disease and Dietary Guidelines for Americans recommendations of 133 and 196 g per week (19 and 28 g per day) [21,22]. Bolded numbers indicate statistical significance at *p* < 0.05.

## Supplementary Materials

The following are available online at https://www.mdpi.com/2072-6643/12/8/2265/s1, Table S1: Estimated hazard ratios for all-cause and 15 cancer incidence and estimated median ages of cancer occurrence fordifferent tertile combinations of intake levels for red and processed meat with vegetables and fruit, whole grains and fiber among men, Alberta’s Tomorrow Project (*n* = 9825). Table S2: Estimated hazard ratios for all-cause and 15 cancer incidence and estimated median ages of cancer occurrence for different tertile combinations of intake levels for red and processed meat with vegetables and fruit, whole grains and fiber among women, Alberta’s Tomorrow Project (*n* = 16,393).
