# Supplementary material for: Co-consumption of Vegetables and Fruit, Whole Grains, and Fiber Reduces the Cancer Risk of Red and Processed Meat in a Large Prospective Cohort of Adults from Alberta’s Tomorrow Project
Source: Nutrients. 2020 Jul 29;12(8):2265. doi: 10.3390/nu12082265 (PMC7468967; doi:10.3390/nu12082265)
Supplement: Supplementary file 1 [file nutrients-12-02265-s001.pdf]

Supplementary Table S1. Estimated hazard ratios[a] for all-cause and 15 cancer incidence and estimated median ages[b] of cancer occurrence for different tertile combinations of intake levels for red and processed meat with vegetables and fruit, whole grains and fiber among men, Alberta's Tomorrow Project (n=9,825)

		All-cause cancers [c]			15 cancers [d]		
		Vegetables & fruit (serving/day)			Vegetables & fruit (serving/day)		
		<2.7	2.7-4.6	>4.6	<2.7	2.7-4.6	>4.6
Red meat (gram/week)							
<282	HR (CI)	**1.31 (1.02-1.67)**	**1.14 (1.01-1.29)**	Ref.	1.25 (0.96-1.63)	1.12 (0.98-1.28)	Ref.
	Age (CI)	**73.44 (71.59-75.34)**	**75.26 (73.25-77.33)**	75.09 (73.12-77.10)	76.03 (73.88-78.23)	78.02 (75.68-80.43)	77.34 (75.09-79.67)
282-498	HR (CI)	**1.29 (1.05-1.59)**	1.14 (0.98-1.32)	1.01 (0.89-1.14)	**1.34 (1.08-1.68)**	1.15 (0.98-1.35)	0.99 (0.86-1.14)
	Age (CI)	**72.10 (70.19-74.07)**	72.60 (70.77-74.47)	73.79 (71.90-75.73)	**74.42 (72.21-76.69)**	74.90 (72.80-77.07)	76.74 (74.52-79.03)
>499	HR (CI)	**1.28 (1.00-1.63)**	1.14 (0.92-1.40)	1.02 (0.79-1.30)	**1.45 (1.12-1.87)**	1.19 (0.95-1.50)	0.98 (0.75-1.29)
	Age (CI)	**71.01 (69.04-73.04)**	74.16 (72.15-76.23)	73.22 (71.43-75.06)	**72.82 (70.59-75.12)**	76.57 (74.25-78.95)	76.06 (73.96-78.23)
Processed meat (gram/week)							
<81	HR (CI)	**1.29 (1.01-1.65)**	**1.14 (1.01-1.29)**	Ref.	1.27 (0.98-1.65)	1.13 (0.99-1.28)	Ref.
	Age (CI)	**73.03 (71.17-74.95)**	**75.23 (73.27-77.24)**	75.64 (73.67-77.67)	75.46 (73.3-77.69)	77.85 (75.58-80.20)	77.91 (75.64-80.25)
81-180	HR (CI)	**1.44 (1.17-1.77)**	**1.28 (1.11-1.48)**	**1.14 (1.01-1.29)**	**1.45 (1.16-1.80)**	**1.25 (1.07-1.46)**	1.08 (0.95-1.23)
	Age (CI)	**73.28 (71.32-75.29)**	**72.44 (70.61-74.32)**	**73.75 (71.87-75.69)**	**75.29 (73.06-77.59)**	**75.00 (72.87-77.20)**	76.46 (74.26-78.73)
>180	HR (CI)	**1.61 (1.28-2.02)**	**1.45 (1.18-1.77)**	**1.30 (1.02-1.66)**	**1.65 (1.29-2.11)**	**1.38 (1.12-1.72)**	1.16 (0.90-1.51)
	Age (CI)	**70.62 (68.76-72.54)**	**74.26 (72.24-76.34)**	**72.80 (71.03-74.61)**	**73.01 (70.85-75.23)**	**76.5 (74.19-78.89)**	75.93 (73.84-78.09)
		Whole grains (serving/day)			Whole grains (serving/day)		
		<0.75	0.75-1.5	>1.5	<0.75	0.75-1.5	>1.5
Red meat (gram/week)							
<282	HR (CI)	1.20 (0.94-1.52)	1.09 (0.97-1.23)	Ref.	1.29 (1.00-1.67)	1.135 (1.00-1.29)	Ref.
	Age (CI)	73.40 (71.51-75.33)	75.06 (73.07-77.09)	75.20 (73.28-77.16)	76.03 (73.85-78.28)	77.72 (75.42-80.09)	77.79 (75.57-80.08)
283-498	HR (CI)	1.07 (0.87-1.30)	1.09 (0.94-1.26)	1.11 (0.98-1.26)	1.20 (0.97-1.48)	1.17 (1.00-1.36)	1.14 (0.99-1.30)
	Age (CI)	72.09 (70.12-74.11)	72.71 (70.88-74.60)	73.60 (71.78-75.47)	74.53 (72.26-76.88)	75.19 (73.07-77.38)	76.38 (74.24-78.57)
>499	HR (CI)	0.95 (0.75-1.20)	1.09 (0.88-1.33)	1.24 (0.97-1.59)	1.11 (0.87-1.43)	1.20 (0.96-1.50)	1.29 (0.99-1.69)
	Age (CI)	72.59 (70.66-74.56)	71.92 (69.99-73.90)	73.98 (72.12-75.88)	74.61 (72.42-76.87)	74.65 (72.40-76.97)	76.56 (74.40-78.77)
Processed meat (gram/week)							
<81	HR (CI)	0.97 (0.76-1.23)	0.98 (0.87-1.11)	Ref.	1.06 (0.82-1.37)	1.03 (0.90-1.17)	Ref.
	Age (CI)	73.77 (71.87-75.71)	74.86 (72.92-76.86)	75.11 (73.18-77.09)	76.28 (74.09-78.54)	77.33 (75.08-79.65)	77.62 (75.39-79.93)
81-180	HR (CI)	1.09 (0.90-1.33)	1.11 (0.97-1.29)	**1.14 (1.01-1.28)**	1.19 (0.96-1.47)	1.15 (0.98-1.34)	1.11 (0.98-1.26)
	Age (CI)	73.10 (71.14-75.11)	72.82 (70.97-74.72)	**73.49 (71.64-75.39)**	75.43 (73.18-77.76)	75.36 (73.21-77.57)	75.99 (73.84-78.2)
>180	HR (CI)	1.24 (0.98-1.56)	**1.26 (1.04-1.54)**	**1.29 (1.02-1.63)**	**1.33 (1.04-1.70)**	**1.28 (1.04-1.59)**	1.23 (0.96-1.59)
	Age (CI)	70.92 (69.03-72.86)	**72.00 (70.08-73.98)**	**74.03 (72.25-75.85)**	**73.11 (70.95-75.34)**	**74.81 (72.56-77.14)**	76.92 (74.82-79.07)
		Fiber (gram/week)			Fiber (gram/week)		
		<121	121-173	>173	<121	121-173	>173
Red meat (gram/week)							

<282	HR (CI)	**1.31 (1.02-1.69)**	**1.14 (1.01-1.30)**	Ref.	1.25 (0.96-1.64)	1.12 (0.98-1.28)	Ref.
	Age (CI)	**73.48 (71.62-75.38)**	**73.78 (71.85-75.77)**	75.94 (73.79-78.15)	75.87 (73.73-78.07)	76.43 (74.18-78.74)	78.11 (75.66-80.64)
283-498	HR (CI)	**1.25 (1.00-1.57)**	1.14 (0.98-1.33)	1.04 (0.92-1.19)	**1.37 (1.08-1.73)**	1.16 (0.98-1.37)	0.98 (0.85-1.13)
	Age (CI)	**72.03 (70.04-74.07)**	71.96 (70.17-73.79)	74.07 (72.18-76.02)	**74.23 (71.95-76.58)**	74.24 (72.18-76.36)	77.01 (74.78-79.30)
>499	HR (CI)	1.20 (0.92-1.57)	1.14 (0.92-1.42)	1.09 (0.84-1.42)	**1.50 (1.13-1.98)**	1.20 (0.95-1.51)	0.96 (0.73-1.27)
	Age (CI)	71.92 (69.62-74.29)	71.68 (69.81-73.60)	74.33 (72.51-76.19)	**73.42 (70.87-76.06)**	73.82 (71.68-76.02)	77.44 (75.29-79.64)
Processed meat (gram/week)							
<81	HR (CI)	**1.30 (1.01-1.69)**	**1.14 (1.00-1.30)**	Ref.	**1.33 (1.01-1.75)**	**1.15 (1.01-1.32)**	Ref.
	Age (CI)	**73.21 (71.34-75.14)**	**73.85 (71.95-75.8)**	76.41 (74.28-78.59)	**75.48 (73.31-77.71)**	**76.24 (74.05-78.51)**	78.84 (76.39-81.37)
81-180	HR (CI)	**1.40 (1.12-1.75)**	**1.29 (1.11-1.50)**	**1.18 (1.05-1.34)**	**1.49 (1.18-1.89)**	**1.28 (1.10-1.50)**	1.10 (0.96-1.25)
	Age (CI)	**72.86 (70.86-74.90)**	**72.11 (70.28-73.98)**	**74.02 (72.13-75.96)**	**74.79 (72.52-77.12)**	**74.44 (72.34-76.61)**	76.90 (74.68-79.19)
>180	HR (CI)	**1.51 (1.18-1.93)**	**1.45 (1.18-1.79)**	**1.40 (1.10-1.80)**	**1.67 (1.28-2.17)**	**1.42 (1.14-1.77)**	1.21 (0.93-1.57)
	Age (CI)	**71.63 (69.48-73.84)**	**71.33 (69.49-73.22)**	**73.93 (72.14-75.77)**	**73.87 (71.41-76.41)**	**73.66 (71.53-75.85)**	77.04 (74.93-79.22)

Abbreviations: CI, confidence interval; HR, hazard ratio.

a. Adjusted for total energy intake, smoking status, alcohol use, family history of cancer, personal history of chronic disease, rural/urban residence, Body Mass Index, physical activity, and education.

b. Adjusted for total energy intake, smoking status, alcohol use, family history of cancer, personal history of chronic disease, rural/urban residence, BMI, physical activity, and age at enrollment.

c. Primary malignant cancers, excluding non-melanoma skin cancer.

d. 15 cancers with possible links to red and processed meat intake, including colorectal, stomach, pancreas, prostate, breast, bronchus/lung, esophagus, kidney, bladder, ovary, endometrium, non-Hodgkin lymphoma, liver and intrahepatic bile ducts, leukemia, other (thyroid, gallbladder and biliary tract, testis, brain).[11]

Supplementary Table S2. Estimated hazard ratios[a] for all-cause and 15 cancer incidence and estimated median ages[b] of cancer occurrence for different tertile combinations of intake levels for red and processed meat with vegetables and fruit, whole grains and fiber among women, Alberta's Tomorrow Project (n=16,393)

		All-cause cancers [c]			15 cancers [d]		
		Vegetables & fruit (serving/day)			Vegetables & fruit (serving/day)		
		<3.2	3.2-5.4	>5.4	<3.2	3.2-5.4	>5.4
Red meat (gram/week)							
<163	HR (CI)	1.19 (0.97-1.45)	1.09 (0.99-1.20)	Ref.	1.11 (0.89-1.37)	1.05 (0.94-1.17)	Ref.
	Age (CI)	74.96 (73.18-76.78)	77.14 (75.18-79.15)	76.13 (74.31-77.99)	77.69 (75.62-79.82)	79.48 (77.23-81.79)	78.15 (76.09-80.27)
163-294	HR (CI)	1.16 (0.98-1.38)	1.08 (0.95-1.23)	1.00 (0.90-1.12)	1.12 (0.93-1.35)	1.06 (0.93-1.22)	1.01 (0.90-1.14)
	Age (CI)	74.00 (72.16-75.88)	75.47 (73.68-77.30)	76.74 (74.89-78.63)	76.57 (74.45-78.76)	77.71 (75.66-79.81)	79.72 (77.55-81.94)
>294	HR (CI)	1.14 (0.93-1.39)	1.07 (0.89-1.28)	1.01 (0.81-1.26)	1.13 (0.91-1.41)	1.08 (0.89-1.31)	1.02 (0.80-1.30)
	Age (CI)	73.69 (71.74-75.69)	74.86 (73.13-76.64)	78.05 (76.15-80.00)	75.58 (73.39-77.84)	77.75 (75.71-79.84)	80.51 (78.32-82.76)
Processed meat (gram/week)							
<37	HR (CI)	1.22 (0.98-1.51)	1.10 (0.99-1.23)	Ref.	1.10 (0.88-1.39)	1.05 (0.94-1.18)	Ref.
	Age (CI)	76.76 (74.80-78.78)	76.87 (75.02-78.77)	77.22 (75.41-79.07)	79.87 (77.56-82.24)	79.53 (77.38-81.73)	79.63 (77.55-81.77)
37-87	HR (CI)	**1.25 (1.05-1.49)**	**1.17 (1.03-1.32)**	1.09 (0.98-1.21)	**1.22 (1.01-1.47)**	**1.16 (1.01-1.33)**	1.11 (0.99-1.25)
	Age (CI)	**74.17 (72.41-75.97)**	**76.28 (74.45-78.17)**	76.30 (74.43-78.22)	**76.70 (74.68-78.79)**	**78.80 (76.68-80.98)**	78.57 (76.43-80.77)
>87	HR (CI)	**1.29 (1.06-1.57)**	**1.24 (1.04-1.47)**	1.19 (0.96-1.47)	**1.342 (1.09-1.65)**	**1.29 (1.07-1.55)**	1.23 (0.98-1.55)
	Age (CI)	**72.38 (70.59-74.22)**	**74.24 (72.49-76.04)**	77.10 (75.26-78.99)	**74.26 (72.24-76.34)**	**76.6 (74.58-78.68)**	79.73 (77.59-81.92)
		Whole grains (serving/day)			Whole grains (serving/day)		
		<0.6	0.6-1.1	>1.1	<0.6	0.6-1.1	>1.1
Red meat (gram/week)							
<163	HR (CI)	0.94 (0.77-1.15)	0.97 (0.88-1.07)	Ref.	1.02 (0.83-1.26)	1.01 (0.91-1.12)	Ref.
	Age (CI)	75.50 (73.66-77.38)	76.28 (74.42-78.18)	76.41 (74.60-78.28)	77.94 (75.82-80.12)	78.62 (76.48-80.81)	78.64 (76.56-80.78)
163-294	HR (CI)	0.98 (0.83-1.15)	0.96 (0.85-1.08)	0.94 (0.84-1.04)	1.10 (0.93-1.32)	1.01 (0.89-1.15)	0.93 (0.83-1.04)
	Age (CI)	74.23 (72.39-76.12)	75.71 (73.87-77.6)	76.37 (74.58-78.20)	76.59 (74.46-78.77)	78.10 (75.99-80.28)	79.21 (77.12-81.35)
>294	HR (CI)	1.02 (0.84-1.23)	0.95 (0.80-1.12)	0.88 (0.71-1.09)	1.20 (0.98-1.47)	1.02 (0.85-1.22)	0.86 (0.69-1.09)
	Age (CI)	73.73 (71.88-75.62)	75.56 (73.71-77.47)	77.46 (75.61-79.35)	75.71 (73.62-77.86)	78.16 (76.01-80.38)	80.17 (78.02-82.38)
Processed meat (gram/week)							
<37	HR (CI)	0.82 (0.66-1.01)	0.90 (0.81-1.00)	Ref.	1.00 (0.80-1.25)	1.00 (0.89-1.12)	Ref.
	Age (CI)	76.46 (74.58-78.39)	77.87 (75.94-79.85)	76.97 (75.17-78.82)	79.20 (77.00-81.45)	80.32 (78.10-82.60)	79.77 (77.66-81.93)
37-87	HR (CI)	0.98 (0.83-1.17)	0.97 (0.85-1.09)	0.95 (0.86-1.05)	**1.21 (1.01-1.46)**	1.12 (0.98-1.27)	1.03 (0.92-1.15)
	Age (CI)	73.90 (72.15-75.70)	75.51 (73.72-77.34)	77.63 (75.71-79.59)	**76.38 (74.35-78.47)**	77.78 (75.73-79.88)	80.11 (77.91-82.37)
>87	HR (CI)	1.19 (0.98-1.44)	1.03 (0.87-1.23)	0.90 (0.73-1.11)	**1.47 (1.19-1.80)**	**1.25 (1.04-1.50)**	1.06 (0.86-1.32)
	Age (CI)	73.49 (71.62-75.41)	74.40 (72.59-76.24)	75.75 (74.03-77.51)	**75.11 (73.01-77.26)**	**77.04 (74.94-79.20)**	78.20 (76.22-80.24)
		Fiber (gram/week)			Fiber (gram/week)		
		<105	105-150	>150	<105	105-150	>150
Red meat (gram/week)							

<163	HR (CI)	1.10 (0.89-1.36)	1.05 (0.94-1.16)	Ref.	1.06 (0.84-1.33)	1.03 (0.92-1.15)	Ref.
	Age (CI)	74.04 (72.24-75.89)	74.99 (73.15-76.88)	76.93 (75.04-78.88)	76.83 (74.73-79.00)	77.05 (74.95-79.21)	79.23 (77.06-81.46)
163-294	HR (CI)	1.15 (0.96-1.38)	1.03 (0.91-1.17)	0.93 (0.84-1.04)	1.13 (0.93-1.38)	1.04 (0.91-1.19)	0.96 (0.85-1.07)
	Age (CI)	72.54 (70.70-74.42)	74.92 (73.15-76.73)	77.10 (75.25-78.99)	75.16 (73.03-77.36)	77.43 (75.38-79.53)	79.78 (77.64-81.98)
>294	HR (CI)	1.20 (0.97-1.49)	1.02 (0.86-1.22)	0.87 (0.70-1.07)	1.21 (0.96-1.51)	1.05 (0.87-1.27)	0.91 (0.73-1.15)
	Age (CI)	72.07 (70.03-74.17)	74.28 (72.51-76.09)	78.48 (76.63-80.37)	74.20 (71.87-76.60)	76.95 (74.89-79.06)	80.95 (78.82-83.13)
Processed meat (gram/week)							
<37	HR (CI)	1.17 (0.94-1.46)	1.08 (0.97-1.21)	Ref.	1.12 (0.87-1.43)	1.06 (0.94-1.19)	Ref.
	Age (CI)	75.40 (73.46-77.38)	76.07 (74.22-77.97)	77.27 (75.46-79.13)	78.38 (76.12-80.72)	78.78 (76.63-81.00)	79.78 (77.68-81.92)
37-87	HR (CI)	**1.26 (1.05-1.52)**	**1.15 (1.01-1.30)**	1.04 (0.94-1.16)	**1.25 (1.02-1.53)**	**1.17 (1.02-1.34)**	1.09 (0.97-1.22)
	Age (CI)	**73.12 (71.32-74.96)**	**74.51 (72.79-76.28)**	78.03 (76.04-80.07)	**76.05 (73.94-78.21)**	**76.50 (74.54-78.50)**	80.61 (78.31-82.97)
>87	HR (CI)	**1.35 (1.10-1.65)**	**1.21 (1.02-1.44)**	1.09 (0.88-1.34)	**1.40 (1.13-1.75)**	**1.29 (1.07-1.56)**	1.19 (0.95-1.49)
	Age (CI)	**70.81 (68.95-72.73)**	**73.92 (72.12-75.76)**	77.24 (75.47-79.05)	**72.66 (70.55-74.83)**	**76.60 (74.51-78.76)**	79.54 (77.51-81.63)

Abbreviations: CI, confidence interval; HR, hazard ratio.

a. Adjusted for total energy intake, smoking status, alcohol use, family history of cancer, personal history of chronic disease, rural/urban residence, Body Mass Index, physical activity, and education.

b. Adjusted for total energy intake, smoking status, alcohol use, family history of cancer, personal history of chronic disease, rural/urban residence, BMI, physical activity, and age at enrollment.

c. Primary malignant cancers, excluding non-melanoma skin cancer.

d. 15 cancers with possible links to red and processed meat intake, including colorectal, stomach, pancreas, prostate, breast, bronchus/lung, esophagus, kidney, bladder, ovary, endometrium, non-Hodgkin lymphoma, liver and intrahepatic bile ducts, leukemia, other (thyroid, gallbladder and biliary tract, testis, brain).[11]
